# Health technology assessment 2025 and beyond: lifecycle approaches to promote engagement and efficiency in health technology assessment

**DOI:** 10.1017/S0266462323000090

**Published:** 2023-02-23

**Authors:** Rebecca Trowman, Antonio Migliore, Daniel A. Ollendorf

**Affiliations:** 1 Health Technology Assessment International (HTAi), Perth, WA, Australia; 2 Health Technology Assessment International (HTAi), Edmonton, AB, Canada; 3 Tufts Medical Center, Institute for Clinical Research and Health Policy Studies, Boston, MA, USA

**Keywords:** health policy, health technology assessment, technology lifecycle, collaboration, stakeholder involvement, transparency

## Abstract

Lifecycle considerations have always been part of health technology assessment (HTA). However, the concept of taking a fuller, more holistic “lifecycle approach” is gaining interest in the HTA community. The 2022 HTAi Global Policy Forum (GPF) discussed how adopting a lifecycle approach could promote stakeholder engagement and robust evidence generation, and whether it could enhance information sharing and transparency across stakeholder groups. This article summarizes the discussions held at the 2022 HTAi GPF and subsequent HTAi Annual Meeting panel session that debated some of the key challenges and opportunities, with particular focus on the pre- and postmarket and disinvestment phase activities. Core themes and recommendations identified that collaboration and patient involvement are happening but still needs to be strengthened, and moving to disease-based approaches may help, although individual contexts still need to be considered. Appropriately developed and mandated core outcome sets may help with information sharing and efficiency in all lifecycle activities. Further, methods for the appropriate use of big data and digital data collection should be developed and driven by the HTA community. The value of lifecycle activities should be reviewed; in particular, scientific advice appears valuable, but the magnitude of effect is somewhat unknown due to the challenges around the confidential nature of these activities. Not all lifecycle activities can be conducted for every technology, and while there is a move away from disinvestment phase activities, more structured prioritization criteria are required. This article ends with suggested next steps to bring forward some of the priority recommendations.

## Introduction

The definition of health technology assessment (HTA) was updated in 2020 and defines HTA as “a multidisciplinary process that uses explicit methods to determine the value of a health technology at different points *in its lifecycle.*” Within this definition, both the intended and unintended consequences of using a health technology versus other alternatives are assessed at different points in the lifecycle of a health technology, which are defined as “pre-market, during market approval, post-market, through to disinvestment” (1). Examples of such methods and activities routinely conducted and/or supported by HTA bodies during these different points in the lifecycle of a health technology (to be termed “lifecycle activities” for the purposes of this article) are provided in Table 1. The focus of discussions at the meeting was on a broad scope of activities that may be undertaken by an HTA body, even if not part of the traditional HTA appraisal process.

**Table 1.** Table of definitions of routinely conducted pre- and postmarket activities

Phase	Activity	Definition
Premarket	Horizon scanning	The systematic identification of health technologies that are new, emerging, or becoming obsolete and that have the potential to affect health, health services, and/or society (HTA Glossary)
	Early dialog/ scientific advice	Scientific advice (or early dialog process) is offered by regulators and/or HTA agencies to companies developing medicines and, increasingly, devices and diagnostics; this may also involve early data synthesis. In some countries, it is conducted as a fee-for-service (2)
Postmarket	Monitoring implementation	Obtaining data to track the uptake of HTA recommendations within a health system, including reimbursement (or not) of technologies having being assessed in the context of a HTA and particularly in the context of being implemented with a managed entry agreement (which can be performance/outcome or financially based) (3)
	Health technology reassessment	A structured, evidence-based assessment of the clinical, social, ethical, and economic effects of a technology currently used in the health system to inform the optimal use of that technology in comparison with alternatives. In some countries, it is conducted as a fee-for-service (4)
	Optimization	Optimization involves assessment or reassessment of a technology, a decision on optimal use, and a decision on implementation (5)
Disinvestment	Disinvestment	The deliberate and systematic reduction of (government) funding for a health technology of questionable or comparatively low clinical and/or economic value (HTA Glossary)

Lifecycle activities (such as those described above) have always been part of the HTA process (6). However, the concept of taking a fuller, more holistic lifecycle approach (as opposed to a more piecemeal conduct of individual lifecycle activities), is currently gaining momentum in the HTA community, evolving to ensure that the needs of various stakeholders continue to be met. While there is no universally accepted definition of the term “lifecycle approach,” elements of a lifecycle approach already exist in HTA practice. For example, where several submissions are made in sequence for a technology as it is developed (e.g., a technology introduced for third-line use becoming approved for second-line use, or a submission for a screening test followed by a submission for diagnosis or as a technology evolves in design or formulation, the latter particularly in the case of devices), the evidence obtained through the first lifecycle activities for that technology may be translated into other lifecycle activities for the same technology, perhaps where it is used in combination with other technologies. This necessitates consideration of whether clinical practice has changed, identification of what data can be reused, and whether the evidence base is therefore reflective of current practice. The main aim of the 2022 HTAi Global Policy Forum (GPF) was therefore to discuss how engagement and information sharing between and within stakeholders throughout the technology lifecycle can be enhanced and made more efficient, and whether the use of a holistic lifecycle approach across the lifecycle of a technology could promote robust evidence generation and transparent communication across all stakeholders. The greatest potential for alignment across the GPF membership was in focusing on “premarket” activities and “postmarket” and disinvestment activities. These activities do not represent two ends of a single timeline but rather two phases of a cycle which is continual/iterative and that will likely evolve. For example, learning from postmarket activities may inform HTA activities and deliberations during a subsequent premarket phase for a similar technology or in the same clinical field. Over 26–28 March 2022, ninety-three representatives from not-for-profit organizations (public HTA bodies, private HTA organizations, payers, and health systems) and for-profit organizations (pharmaceutical, biotech, and device companies), patient representatives, invited speakers, and HTAi leadership met in a hybrid format to discuss these issues.

This article summarizes the discussions held at the 2022 HTAi GPF. The meeting was conducted under the Chatham House Rule (7), whereby participants are free to share information obtained at the meeting, but they may not reveal the identity or affiliation of the person providing the information. This is then supplemented with a summary of the discussion at the panel session held at the 2022 HTAi Annual Meeting. This article presents the authors’ view on the 2022 GPF and is not a consensus or official statement from individuals who attended the meeting or their organizations.

## GPF meeting structure

To inform the meeting discussion and activities, a background paper was developed (8), which presented an overview of the topic, current contextual issues, results of a literature review, and details of lifecycle activities currently undertaken or planned by various HTA bodies worldwide.

The GPF consisted of a mix of presentations, case studies and a panel session representing HTA bodies, regulators, patient organizations, industry, and payer perspectives to stimulate debate on the key issues. This was followed by breakout sessions where smaller groups discussed challenges and barriers, opportunities, and next steps related to six key themes. Breakout themes were identified through thematic analysis of the conclusions and recommendations from the previous GPF documents, supplemented by interviews with a wide range of expert informants. A summary of the themes is listed below and described in more detail in Supplementary Table 1.

1. Collaboration within and between HTA bodies and with the wider health ecosystem
2. Data generation and information preservation
3. Standardizing evidence requirements and process frameworks
4. Staff and skills shortages: resourcing lifecycle activities in HTA
5. Engaging stakeholders in lifecycle activities in HTA: patients, payers, clinicians, and beyond (e.g., caregivers, society)
6. Transparency and consistency in HTA policies, procedures, and outputs.

These discussions were followed by a multistakeholder panel session at the HTAI Annual Meeting (held 24–29 June 2022 in the Netherlands) that included a moderated audience question and answer session. The discussions from the GPF have been synthesized with the annual meeting panel session debate, and the results are presented according to the key themes below. Pertinent new examples were not included in the GPF background paper due to timing of their publication – for example, the lifecycle HTA framework by Kirwin et al. (9) that describes a novel lifecycle approach to technology assessment incorporating the lifecycle activities previously defined but with a sequential implementation and embedded decisions rules, based on evidence generation and risk-based pricing strategies.

## Discussion results by theme

### Collaboration within and between HTA bodies and with the wider health ecosystem

In taking a lifecycle approach to HTA, efficient and coordinated heath systems are required to enable the sharing of data, learnings, and flexible and adaptive responses to new evidence; all of which require collaboration. Collaborative approaches both across HTA bodies and beyond with regulators, payers, and other health system stakeholders are underway; however, siloes persist across the health evidence ecosystem (10). These siloes often result in researchers generating evidence for questions already answered or questions that are not priorities for decision-makers and create challenges in sharing data and communication barriers (11). The existing siloes need to be disbanded or lowered, and we need to act in more engaged, systematic, efficient, and coordinated ways across all stakeholder groups and organizations with the patient at the center. In particular, the HTA community and health researchers should work together earlier in the technology lifecycle to better set research priorities and routinely involving patients when establishing research agendas. Additionally, greater collaboration between HTA bodies and health system stakeholders on monitoring the appearance of relevant new evidence and utilization patterns of technologies is worthy of greater attention with greater sharing of clinical data and HTA learnings (12). This could include reviewing the performance of managed entry agreements (MEAs) and methods on a global scale to ensure that administratively burdensome and ineffective MEAs are phased out, and information collected in the context of MEAs could be better shared globally. Horizon scanning is best done collaboratively at a regional (e.g., between European countries) or an international level rather than a local national level (13).

Collaborations have the potential to lead to more aligned, efficient, and equitable healthcare for patients; however, the shift in perspective required for collaborative work such as shared goals, beliefs, and values is often the most challenging aspect (14). While collaboration is likely to be most efficient where processes are similar, a key requirement for any collaboration is the development of trust between stakeholders and organizations and building this requires resources and effective leadership may take many years (15;16). The introduction of the EU HTA Regulation, for example, is a culmination of over 25 years of work (14;17) and will be closely monitored to understand whether the efforts are truly aligned and increase efficiency.

The recently announced AUS-CAN-UK HTA collaboration arrangement (18) is another example of a cross-jurisdictional arrangement between HTA bodies that will aim to work together to address mutually agreed priority areas such as interaction with regulators and the use of digital health and artificial intelligence. Other examples represent increasingly open dialogs and collaborations between HTA bodies and regulators, many of which also include industry, payers, patients, and other stakeholders. Many of these were summarized by the 2021 Centre for Innovation in Regulatory Science report (19), and key examples include Project Orbis and the Innovative Licensing Access Pathway (ILAP) in the UK. Project Orbis is a global collaborative review program that was initiated by the US Food and Drug Administration to provide a framework for concurrent submission and review of oncology products among international regulatory authorities with involvement from HTA bodies. The ILAP aims to deliver safe, early, and financially sustainable patient access to innovative medicines using an integrated pathway pulling together expertise from across the UK regulator, HTA bodies, and healthcare system payers. National and international collaboration and priority setting, while maintaining independent guidance, is necessary to implement holistic lifecycle approaches and continuing to inform fair resource allocation across health systems and societies. The HTAi position statement taskforce (as described later in this article) will maintain a watching brief on key collaborations such as these.

### Data generation and information preservation

The continual generation of data underpins lifecycle approaches; without this, recommendations cannot be reassessed, and thus, various lifecycle activities cannot be undertaken. Data can be difficult to obtain, but ironically, we can also be overwhelmed by the amount of available data. Early engagement and collaboration with all stakeholders can help to start conversations that help to refine the demands on evidence and can help ensure efficiency in evidence generation and data collection (20). Adopting a holistic lifecycle approach can enhance this aspect further and enable better planning for the evaluation of the value of reassessing or adding to the evidence base for a given technology with the aim of reducing resolvable uncertainty, as noted in the 2021 HTAi GPF (21). Digital data collection and use of advanced big data methods are key advancements that should enable generation and sharing and analysis of new evidence. Exploration of how to better gather and share these data and development of methods and frameworks is needed to guide the use of real-world data (RWD) and real-world evidence (RWE). Importantly, the HTA community should play a pivotal role in this, in partnership with others and building on what already exists.

The development and increased use of core outcome sets (an agreed standardized set of outcomes that should be measured and reported as a minimum in all clinical trials of a specific condition) represent an opportunity for increasing efficiency of HTA conduct when taking a lifecycle approach (22). The development of core outcome sets internationally could be a way to make early engagement and data generation more efficient; appropriately developed core outcome sets (i.e., including clinician and patient input) can potentially be used at all stages of the technology lifecycle to guide data generation plans. Examples of such outcome sets include the Core Outcome Measures of Effectiveness in Trials initiative that aims to foster and facilitate methodological research in the area of standardizing outcomes (22) and the International Consortium for Health Outcomes Measurement that aims to define global sets of patient-centered outcome measures that matter most to patients (23).

Another recent example highlighted at the panel session was the European Innovative Medicines Initiative Health Outcomes Observatories (H2O). The H2O intends to provide patients with digital tools (including an app) to report their core health outcomes in a standardized way. Patients will maintain control of their own data and will decide who can access it. The data will be integrated with traditionally collected information (e.g., electronic health records, payer databases) and used to inform individual patient care but also anonymized to allow aggregation and comparison across disease areas and technology lifecycles. Long-term, multistakeholder discussions built on mutual trust are required to build these extensive observatories. Actions that help increase the development and use of core outcome sets should continue to be explored by HTA bodies and others; this could reduce duplication of effort, allow greater sharing of data sets across jurisdictions, and set the future research agenda for all individual technologies within a given disease.

### Standardizing evidence requirements and process frameworks

Evidence requirements and process frameworks (both across HTA bodies and across HTA bodies and other health system stakeholders such as regulators) should be standardized wherever possible to facilitate the sharing of information that is needed in taking a lifecycle approach. While topics, methods, and outputs of HTA bodies will vary due to their various roles and remits, building consistent frameworks could allow HTA bodies to choose relevant items to be applied in the local context. Examples of the outcomes of European HTA were presented, suggesting that assessments that result in different recommendations often have similar content, and the HTA community could save time working more closely with each other and regulators (24). Further, key areas that could be supported by the development of frameworks and standards include but are not limited to: HTA topic selection and priority setting; use of RWD and RWE over the technology lifecycle approaches, and adoption of standards to ensure RWD is reliable; and use of MEAs and impact on pricing changes, for example, prices going up as well as down reflecting additional value in greater certainty on a technology.

There is a need to develop selection criteria for targeted projects and then share oversight, data systems and analysis with strong involvement from all stakeholders. A baseline understanding of evidence requirements and process frameworks, applied more uniformly, could also assist with acute resource constraints and shortfalls. In parallel, greater transparency in activities such as scientific advice should also be explored in a predictable way for all stakeholders. This could also be supported by developing more consistent frameworks and standards, which could allow current activities to be made more flexible and agile.

### Staff and skills shortages: resourcing lifecycle activities in HTA

The demands on HTA bodies are extensive and are notably increasing with limited staff and budgets available, and this is a key concern when considering which (if any) lifecycle activities can be undertaken and whether a lifecycle approach is feasible. Collaborating, sharing workloads, promoting internal talent, and providing training opportunities for staff are vital for the sustainability of HTA bodies. Prioritization is needed to determine where lifecycle activities could add the most value; one example is in the case of technologies being developed for populations with an unmet medical need, particularly pertaining to accelerated regulatory approval, where urgent clinical need is most acutely juxtaposed with technologies that come with a very high list price at launch. HTA methods and processes, however, should not change for every new technology that is assessed; rather, flexibility and agility are required to produce adaptive, fit-for-purpose approaches that maximize the efficiency of staff and resources. For example, a less intensive scientific advice process could be developed for certain technologies and conditions.

A prerecorded case study presentation was available before the GPF that highlighted the experience of Health Technology Wales (About – Health Technology Wales), a HTA body established with a lifecycle approach embedded within its remit. In this presentation, resource constraints were clearly acknowledged, but it was also emphasized how much is possible for one small HTA body to achieve through planning, collaborating, and being appropriately integrated into a health evidence ecosystem. In addition, numerous examples of skills that need to be developed within the HTA community were highlighted. For example, the use of RWE is a key element in a lifecycle approach, and new skill sets (e.g., in curation, governance and analysis) are needed. Potential greater involvement in postmarket activities would require HTA bodies to have staff with skills beyond evidence synthesis including collecting and analyzing observational data and greater health systems and policy experience, given the policy issues associated with HTA decisions and their implementation.

### Involving stakeholders in lifecycle activities in HTA: patients, payers, clinicians, and beyond (e.g., caregivers, society)

Stakeholder involvement is a critical component of every HTA lifecycle activity. Early stakeholder input and dialog has the potential to influence clinical trial development programs to focus more on patient needs, and this could contribute to reduced clinical trial timelines and costs and reduced time to patient access. In particular, it is critical to meaningfully incorporate the patient and, where appropriate, caregiver perspectives into premarket activities (25). While very early involvement is potentially challenging given limited available data and some technologies not progressing to regulatory approval, better involvement of patients and clinicians in disease area-specific technology development and identifying where there is clinical unmet need could be valuable. Patients also need to be involved in a meaningful way throughout the HTA process; to do so, they should be appropriately compensated for their input and offered training. Further, repositories of patient information could be created globally to avoid duplication of effort (26;27).

Payers also need to be engaged early; however, this is unlikely, and probably unnecessary, for every technology. For example, one solution may be to establish regular advisory committees with payer representation for the purposes of horizon scanning or early advice, particularly to focus on potentially high-cost technologies (28). Finally, including the technology manufacturers early in HTA discussions may also be beneficial, with dialog to help HTA agencies understand the development plans and for the manufacturer to fulfill HTA needs when preparing and optimizing clinical development. In these premarket activities however, care must be taken to avoid formulating possible barriers related to the technology in question prematurely.

Patient advocates and clinical experts are increasingly and routinely asked to provide input at various stages of the lifecycle of health technologies. While this is universally accepted as good practice, the current input requirements were noted as stretching the capacity of experts (particularly patients) to meaningfully participate. This is exacerbated by the duplication in input requirements from regulators, industry, HTA bodies, and other stakeholders. Further, there are an increasing number of complex technologies requiring specific technical expertise to evaluate their value. Evolving to a more disease/condition focus rather than technology-specific engagements could increase efficiency and allow, for example, scientific advice to be delivered to multiple technology manufacturers at once.

The example of myasthenia gravis was presented, as a rare neuromuscular autoimmune disease for which multiple companies are developing potential technologies (29). This example highlighted the benefits in the early access, including potential for standardization of outcomes (including patient reported outcomes), sharing patient input across technology developers, and emergence of discussions to build a single disease registry, although it was noted that the data in early phases of technology development are often the property of the manufacturer, posing a potential barrier to sharing data. Caution was, however, advised regarding losing such nuances as patient input and experience relevant to specific technologies.

### Transparency and consistency in HTA policies, procedures, and outputs

Transparency and consistency are important elements that can facilitate a lifecycle approach; having these elements in place will allow data and information sharing within and across jurisdictions. Among HTA bodies, there appears to be more concordance during the premarket phases of the technology lifecycle and less concordance in the postmarket and disinvestment phases. In particular, the opportunities for HTA bodies to achieve a consistent understanding during early formal and informal dialogs and horizon scanning were seen as valuable. The ability to clearly articulate the value and impact of such activities is constrained by the confidential nature of many of these discussions. In part because of this, there is no accepted consensus on performance metrics for scientific advice (30).

While the importance of effective collaboration was noted, this also requires appropriate degrees of transparency in the inputs and outputs of HTA data and processes subject to varying regulations and other conditions from country to country. Whether greater global alignment on transparency is feasible or necessary tends to focus on economic issues and pricing, due to market and funding differences, rather than clinical data where the benefits of effective collaboration could be initially demonstrated. Elevating and aligning the transparency and consistency of the processes across HTA bodies could ultimately lead to greater collaboration. To do this, structured dialog, culture shifts, effective and efficient data infrastructures, and digital systems are required. Further, considering why information is confidential and more closely examining where data can be shared within and across HTA bodies and with other stakeholders may add value in this regard and facilitate meaningful collaboration across HTA bodies and beyond.

The examples discussed at the panel session also highlighted the importance of sharing data and acknowledged the issues discussed at the GPF with data shared “in confidence” (particularly regarding early scientific advice). Recent actions, such as the announcement in May 2022 by the International Committee of Medical Journal Editors that stated “*the ICMJE does not consider results or data contained in assessment reports published by health technology assessment agencies, medical regulators, medical device regulators, or other regulatory agencies to be duplicate publication*” (31), were highlighted as steps toward greater transparency, particularly of unpublished clinical data. Other steps that were suggested during the panel session included allowing sharing of appropriately masked or deidentified confidential data with patient representatives to facilitate their input. It was also noted that while there is international intent for greater sharing of data sets, particularly pertaining to rare diseases, economic models and MEAs pose competitive and proprietary challenges to execution of this ideal.

## Summary

The discussions at the GPF and subsequent HTAi Annual Meeting panel session were far-reaching, reflecting the broad nature of the topic selected. However, the key themes and high-level recommendations that emerged were

- HTA bodies and stakeholders should review the value of undertaking lifecycle activities and the problems they are intended to address. Not all lifecycle activities can be conducted for every technology; it is necessary to develop criteria to determine where and for whom such activities can yield the most value. The design and further implementation of lifecycle approaches should reinforce and not impede innovation and patient access to the right technologies at the right time.
- Viewing the lifecycle of a technology through the lens of the value offered by the technology at the various timepoints can enable focusing resources where they are most needed. The HTA community should be flexible and agile in its approach but should not expect to shoulder the workload alone.
- Scientific advice should continue; however, this requires reflecting on how it is conducted and where efficiencies in information sharing could be gained. Among the challenges are maintaining confidentiality and firewalls while disentangling information that can be made public (e.g., on unmet medical needs and end point selection) from information that is commercially sensitive. Such a review at this important phase is essential to maximize the efficient use of HTA and other resources.
- Horizon scanning is best done collaboratively at a regional (e.g., among European countries) or an international level rather than a local national level (13).
- There has been less activity in postmarket HTA, and there seems to be less emphasis on routine health technology reassessment (HTR) and active disinvestment. This may be due partly to the pace of innovation, the remits of HTA bodies, and greater emphasis on premarket HTA phases. One important caveat, however, is that there are technologies for which monitoring is important, for example, high-risk implantable devices, technologies with accelerated regulatory approval, or technologies approved with a MEA, and therefore, *targeted* HTR is appropriate in cases such as these.
- Rather than conducting HTR of individual technologies during their postmarket phases, reassessments could be conducted while developing clinical guidelines on entire diseases and treatment pathways. This is, however, methodologically challenging and collaboration outside of the HTA community is required.
- Collaboration is already underway and could expand on such efforts as the EU HTA Regulation and initiatives such as ILAP in the UK, and in the field of horizon scanning. Setting expectations and opening conversations early are needed across multiple stakeholder groups. Collaboration with external stakeholders in the postmarket HTA phases is important and need to be strengthened.
- Developing and mandating the use of core outcome sets could help to inform sharing and efficiency for HTA bodies, particularly around early scientific advice, and should be encouraged throughout the lifecycle. Core outcome sets should be developed in partnership with patients and clinicians.
- Patient involvement in HTA is essential and must be improved. It should be broad, routine, scalable, and compensated. Exploration of ways to reduce duplication of patient input across stakeholder groups is needed; one option may be to move toward disease-specific rather than technology-specific repositories of patient insight and experience.
- Digital data collection and use of advanced big data methods should enable generation of new evidence, and the possibility of shared, public data sets should be increasingly explored. In partnership with others, the HTA community should play a pivotal role in advancing methods and frameworks for guiding the use of RWD and RWE.(32)

## Limitations

This article represents a summary of discussions held at the HTAi GPF and subsequent panel session at the HTAi Annual Meeting. While these discussions represent a broad range of views and perspectives, the membership of the GPF is such that the majority of the perspectives are from countries with established, mature HTA systems. While informants from beyond the GPF membership were approached for input to the background paper prior to the meeting, this is a limitation of the discussion summary, as emerging and nascent HTA system priorities and potential for adopting a lifecycle approach will differ.

The GPF primarily comprises HTA body representatives and life science industry organizations. Patient representatives are specifically consulted during the development of the background paper and are invited to the meeting to present and participate; however, some key stakeholder groups (such as clinicians, payers, and policy makers) are less well represented in the discussion.

## Discussion and next steps

GPF participants agreed that a position statement on the topic should be developed by a multistakeholder taskforce. The purpose will be to outline the benefits of adopting certain lifecycle activities, the areas of greatest priority for the HTA community, and the steps required to pursue them. The statement will not be a specific set of instructions but will include examples illustrating how lifecycle activities might be prioritized or restricted.

## Supplementary material

For supplementary material accompanying this paper visit https://doi.org/10.1017/S0266462323000090. click here to view supplementary material
