# Supplementary figures and images for: Health technology assessment 2025 and beyond: lifecycle approaches to promote engagement and efficiency in health technology assessment
Source: Int J Technol Assess Health Care. 2023 Feb 23;39(1):e15. doi: 10.1017/S0266462323000090 (PMC11574536; doi:10.1017/S0266462323000090)

**SUPPLEMENTARY MATERIAL**

Supplementary Table 1 - Breakout themes of the Global Policy Forum 2022


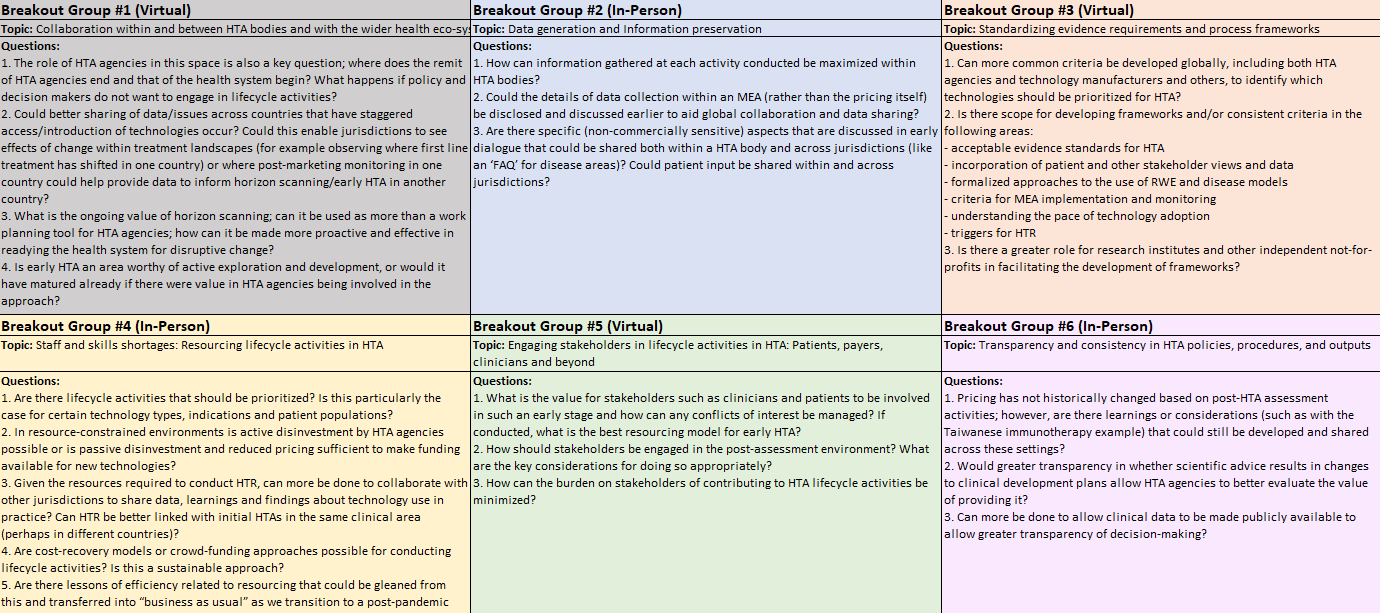

Supplement: Supplementary file 1 [file S0266462323000090sup001.docx]
